# Newborn Screening for Sickle Cell Disease in Catalonia between 2015 and 2022—Epidemiology and Impact on Clinical Events

**DOI:** 10.3390/ijns10040069

**Published:** 2024-10-03

**Authors:** José Manuel González de Aledo-Castillo, Ana Argudo-Ramírez, David Beneitez-Pastor, Anna Collado-Gimbert, Francisco Almazán Castro, Sílvia Roig-Bosch, Anna Andrés-Masó, Anna Ruiz-Llobet, Georgina Pedrals-Portabella, David Medina-Santamaria, Gemma Nadal-Rey, Marina Espigares-Salvia, Maria Teresa Coll-Sibina, Marcelina Algar-Serrano, Montserrat Torrent-Español, Pilar Leoz-Allegretti, Anabel Rodríguez-Pebé, Marta García-Bernal, Elisabet Solà-Segura, Amparo García-Gallego, Blanca Prats-Viedma, Rosa María López-Galera, Abraham J. Paredes-Fuentes, Sonia Pajares García, Giovanna Delgado-López, Adoración Blanco-Álvarez, Bárbara Tazón-Vega, Cristina Díaz de Heredia, María del Mar Mañú-Pereira, José Luis Marín-Soria, Judit García-Villoria, Pablo Velasco-Puyó, on behalf of the Sickle Cell Disease Newborn Screening Group of Catalonia

**Affiliations:** 1 Section of Inborn Errors of Metabolism, Department of Biochemistry and Molecular Genetics, Hospital Clínic de Barcelona, 08028 Barcelona, Spain; argudo@clinic.cat (A.A.-R.); rmlopez@clinic.cat (R.M.L.-G.); paredes@clinic.cat (A.J.P.-F.); spajares@clinic.cat (S.P.G.); delgado@clinic.cat (G.D.-L.); jlmarinsoria@gmail.com (J.L.M.-S.); jugarcia@clinic.cat (J.G.-V.); 2 Hematology Department, Hospital Universitari Vall d’Hebron, 08035 Barcelona, Spain; david.beneitez@vallhebron.cat (D.B.-P.); adoracion.blanco@vallhebron.cat (A.B.-Á.); barbaraymaine.tazon.vega@psmar.cat (B.T.-V.); 3 Pediatric Oncology and Hematology Department, Hospital Universitari Vall d’Hebron, 08035 Barcelona, Spain; anna.colladogimbert@vallhebron.cat (A.C.-G.); cristina.diazdeheredia@vallhebron.cat (C.D.d.H.); pablo.velascopuyo@vallhebron.cat (P.V.-P.); 4 Pediatric Hematology Unit, Hospital Germans Trias i Pujol, 08916 Badalona, Spain; falmazan.germanstrias@gencat.cat; 5 Pediatric Department, Hospital Santa Caterina, Institut d’Assistència Sanitària, 17190 Salt, Spain; silvia.roig.ias@gencat.cat (S.R.-B.); anna.andres.ias@gencat.cat (A.A.-M.); 6 Pediatric Oncology and Hematology Department, Hospital Sant Joan de Déu, 08950 Barcelona, Spain; anna.ruiz@sjd.es (A.R.-L.); georgina.pedrals@sjd.es (G.P.-P.); 7 Pediatric Hematology Unit, Hospital Universitari Sant Joan de Reus, 43204 Reus, Spain; dvd.medina1@gmail.com; 8 Pediatric Department, Hospital Universitari Arnau de Vilanova, 25198 Lleida, Spain; gemmanadalrey@gmail.com (G.N.-R.); marinaespisal@gmail.com (M.E.-S.); 9 Pediatric Hematology Unit, Hospital General de Granollers, 08402 Granollers, Spain; mcoll@fphag.org; 10 Pediatric Department, Hospital de Figueres, 17600 Figueres, Spain; marcelinalgar@hotmail.com; 11 Pediatric Oncology and Hematology Department, Hospital de Sant Pau, 08041 Barcelona, Spain; mtorrent@santpau.cat; 12 Hematology Department, Hospital de Sant Pau, 08041 Barcelona, Spain; pleoz@santpau.cat; 13 Pediatric Hematology Department, Consorci Sanitari del Maresme, 08304 Mataró, Spain; arodriguez969@csdm.cat; 14 Pediatric Hematology Department, Consorci Sanitari de Terrassa, 08227 Terrassa, Spain; mgarcia@mutuaterrassa.es; 15 Pediatric Hematology Department, Hospital Universitari Mútua de Terrassa, 08221 Terrassa, Spain; 16 Institut Català de la Salut (ICS) Catalunya Central, 08500 Vic, Spain; esolas.cc.ics@gencat.cat (E.S.-S.); garciagallego.amparo@gmail.com (A.G.-G.); 17 Maternal and Child Health Service, Public Health Agency of Catalonia (APSCAT), Department of Health, Generalitat de Catalunya, 08005 Barcelona, Spain; blanca.prats@gencat.cat; 18 Center for Biomedical Research Network on Rare Diseases (CIBERER), ISCIII, 28029 Madrid, Spain; 19 Biomedical Research Institute, August Pi i Sunyer (IDIBAPS), 08036 Barcelona, Spain; 20 Rare Anemia Disorders Research Laboratory, Cancer and Blood Disorders Research Group, Vall d’Hebron Institut de Recerca (VHIR), 08035 Barcelona, Spain; mar.manu@vhir.org

**Keywords:** sickle cell disease, hemoglobinopathies, newborn screening, clinical events, treatment intervention

## Abstract

In 2015, Catalonia introduced sickle cell disease (SCD) screening in its newborn screening (NBS) program along with standard-of-care treatments like penicillin, hydroxyurea, and anti-pneumococcal vaccination. Few studies have assessed the clinical impact of introducing NBS programs on SCD patients. We analyzed the incidence of SCD and related hemoglobinopathies in Catalonia and the change in clinical events occurring after introducing NBS. Screening 506,996 newborns from 2015 to 2022, we conducted a retrospective multicenter study including 100 screened (SG) and 95 unscreened (UG) SCD patients and analyzed SCD-related clinical events over the first six years of life. We diagnosed 160 cases of SCD, with an incidence of 1 in 3169 newborns. The SG had a significantly lower median age at diagnosis (0.1 y vs. 1.68 y, *p* < 0.0001), and initiated penicillin prophylaxis (0.12 y vs. 1.86 y, *p* < 0.0001) and hydroxyurea treatment earlier (1.42 y vs. 4.5 y, *p* < 0.0001). The SG experienced fewer median SCD-related clinical events (vaso-occlusive crisis, acute chest syndrome, infections of probable bacterial origin, acute anemia requiring transfusion, acute splenic sequestration, and pathological transcranial Doppler echography) per year of follow-up (0.19 vs. 0.77, *p* < 0.0001), a reduced number of annual emergency department visits (0.37 vs. 0.76, *p* < 0.0001), and fewer hospitalizations (0.33 vs. 0.72, *p* < 0.0001). SCD screening in Catalonia’s NBS program has effectively reduced morbidity and improved affected children’s quality of life.

## 1. Introduction

Sickle cell disease (SCD) constitutes a group of the most prevalent structural hemoglobinopathies [1]. SCD is characterized by a point mutation in the *HBB* gene, which encodes the substitution of glutamate to valine at the sixth amino acid residue of the β-globin chain. This substitution produces the sickle hemoglobin allele β^s^ (HbS), forming a sickle β-globin subunit. SCD presents with three main genotypes, which are sickle cell anemia (SCA), where the β^s^ allele is present in homozygosity (HbSS); a form where the β^s^ allele is in compound heterozygosity with hemoglobin C (HbSC); and one where the β^s^ allele combines with either a β^0^ or β^+^ allele (HbSβ-thalassemia) [2]. While there have been descriptions of other genotypes that can cause SCD, most of these are rare [3].

Patients with SCD experience chronic organ damage and acute vaso-occlusive crisis (VOC), which can cause pain and organ failure, such as acute chest syndrome (ACS) [2]. Hyposplenism, induced by progressive ischemic organ damage, is a particularly significant consequence, as it increases the susceptibility to infections [4]. This is a primary cause of morbidity and mortality in children under five years of age in low-income countries.

In 2021, the estimated annual global birth incidence of SCD was 515,000, with approximately 79% occurring in sub-Saharan Africa. The SCD mortality corresponds to up to 8.4% [5] of all-cause mortality in Africa for under-five-year-olds, with the under-five SCD mortality in this region estimated to be up to 90% [6]. In Europe, there are far fewer cases, with an estimated 28,821 to 48,928 SCD patients living in the 12 EU member states that contribute to the Rare Anemia Disorders European Epidemiological Platform (RADeep), which are Belgium, Cyprus, Denmark, France, Germany, Greece, Ireland, Italy, Portugal, Spain, Sweden, and The Netherlands [7]. Data from the Spanish National Registry collected until 2023 show a prevalence of 1.317 patients with SCD in Spain. However, this number may be underestimated due to potential exclusions of deceased patients or those lost to follow-up, as well as the recent participation of adult hematologists in the registry [8]. The under-five mortality rate attributed to SCD in Spain is 2.8% [5].

The first newborn screening (NBS) program for SCD was introduced in New York in 1975 [9]. Across Europe, many countries have since established NBS programs for SCD. Some are national programs, some are regional, and some target specific ethnic groups [10,11,12,13,14]. Recently, the American Society of Hematology (ASH) established the Consortium on Newborn Screening in Africa (CONSA) for SCD to implement standardized hemoglobinopathy NBS and early interventions for children with SCD in seven countries across sub-Saharan Africa [15,16]. Spain added SCD to its national mandatory NBS panel in 2013, with some regions starting earlier [17]. Catalonia began a targeted pilot study in 2013 and actively began universal screening in 2015 [18].

Few studies have investigated the effect of NBS on the occurrence of clinical events in children with SCD. The Belgian NBS program for SCD compared two cohorts, one diagnosed through NBS in the regions where NBS for SCD is available, and the other diagnosed through healthcare providers after presenting symptoms in regions where NBS is not available. The study concluded that the screened group had a higher survival rate without bacteremia, as well as lower hospitalization rates [19]. The NBS program of Madrid has recently published the impact of the NBS of SCD on clinical events, stroke prevention, and survival [20]. However, no existing study has assessed the effect of NBS on the most frequent SCD-related events.

This study has two primary objectives. The first is to assess the incidence of SCD and other hemoglobinopathies in Catalonia following the implementation of NBS and to describe the populational epidemiological characteristics. The second objective is to evaluate the impact of NBS on the diagnosis, treatment, and occurrence of clinical events in SCD patients.

## 2. Materials and Methods

### 2.1. Catalonian NBS Program 

From January 2015 to December 2022, Catalonia’s NBS program analyzed 506,996 dried blood spot (DBS) samples corresponding to all newborns in Catalonia over that period and screened them for SCD. The recommended age at sample collection was 48–72 h after birth. Newborns who received a blood transfusion before the sample was taken were scheduled three months after the last transfusion for a new sample extraction. Demographic data were electronically collected from the NBS database. Each day, DBS samples collected from hospitals across Catalonia were transported to the NBS laboratory at the Hospital Clínic de Barcelona, where they were analyzed.

The Catalonian NBS program aims to identify patients with the most severe phenotypes of SCD, namely HbSS, HbSC, HbSβ^0^, and HbSβ^+^. However, the capillary electrophoresis (CE) technique used also enables the detection of other types of hemoglobin such as fetal hemoglobin (HbF), hemoglobin D (HbD), hemoglobin E (HbE), hemoglobin OArab (HbOArab), hemoglobin Korle-Bu (HbKorle-Bu), and other less common variants [21]. In addition, the technique identifies carriers of the β^s^, β,^c^, β^D^, and β^E^ alleles, as well as patients with suspected HbH disease and beta thalassemia major, transfusion-dependent thalassemias, and gamma thalassemia. Detected cases are referred to the Clinical Reference Unit (CRU) at Hospital Universitari Vall d’Hebron, and treatment programs are initiated as necessary.

Hemoglobin pattern analysis in dry blood spot (DBS) samples (3.8 mm diameter spot) was conducted using capillary electrophoresis (CE) with the commercial kit designed for the Sebia Capillarys 2 system (Sebia Inc., Lisses, France). Before capillary injection, DBS specimens underwent onboard hemolysis and were separated based on electrophoretic mobility [21]. Quantification of eluted fractions was conducted spectrophotometrically at a wavelength of 415 nm, and the peaks were identified based on their migration within predefined zones using the Phoresis 8 software (Sebia Inc., Lisses, France).

Positive cases of SCD and other hemoglobinopathies were referred to the CRU in the Pediatric Oncology and Hematology Department at the Hospital Universitari Vall d’Hebron, Barcelona. Here, families were scheduled for confirmatory analysis and clinical follow-up with the reference pediatric hematologist when the infant reached two months of age. The confirmatory analysis was conducted using high-performance liquid chromatography (HPLC) with the Variant II-Beta-Thalassemia Short Program^®^ (Bio-Rad, Hercules, CA, USA). This method can detect hemoglobin F (HbF), hemoglobin A (HbA), HbS, HbC, HbD, HbE, and other variants in a procedure that takes 6.5 min, offering high sensitivity and specificity. When necessary, electrophoresis techniques were also carried out (alkaline pH and acidic pH), performed by the Hydrasys System (Sebia Inc.,Lisses, France).

Additionally, genetic analyses were performed in newborns, which include sequencing of the complete *HBB* gene via Sanger sequencing. The most common alpha-globin mutations were assessed through polymerase chain reaction (PCR) analysis and reverse hybridization using the α-Globin StripAssay® (ViennaLab Diagnostics, Vienna, Austria) [22]. These tests were conducted simultaneously using the same blood specimen. In case of any discrepancy or inconclusive result, a multiplex ligation-dependent probe amplification (MLPA) of beta and alpha clusters was performed [23,24]. Moreover, parents and siblings were screened if they had not previously undergone screening.

**Figure 1:**
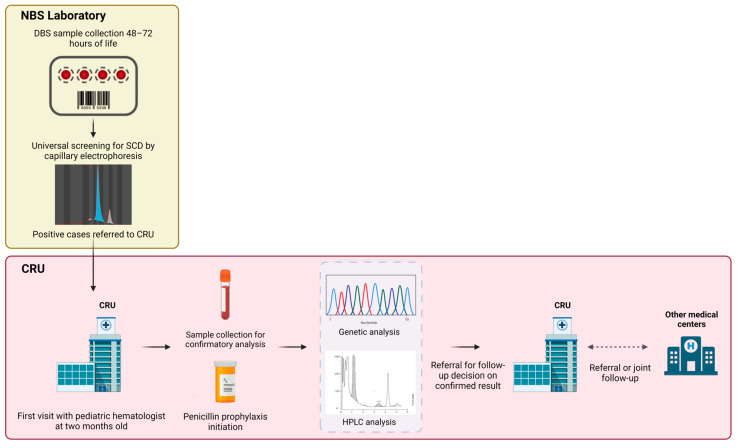
Catalonian NBS process. DBS: dried blood spots; SCD: sickle cell disease; CRU: Clinical Reference Unit; HPLC: high-performance liquid chromatography.

The standard of care for SCD patients diagnosed by NBS adheres to the “Sociedad Española de Hematología y Oncología Pediátricas” (Spanish Society of Pediatric Hematology and Oncology—SEHOP) 2019 consensus [25]. This comprehensive care approach includes measures such as antibiotic prophylaxis and vaccinations targeting encapsulated pathogens, among other interventions. In addition, parents receive training on how to care for a child with SCD and are provided with informative materials about the condition. They are also given contact details for the CRU and information about patient associations. Subsequent patient follow-up takes place within a multicentric and interdisciplinary network. Follow-up consultations occur every 3–4 months for the HbSS and HbSβ^0^ genotypes, and every 6 months for the HbSC and HbSβ^+^ genotypes. Patients receive treatment at medical centers with varying levels of complexity, tailored to their individual needs. All coordination is managed by the CRU. One of the strong recommendations of the CRU to medical centers is to initiate treatment with hydroxyurea at nine months of age in patients with homozygous HbSS and HBSβ^0^ thalassemia genotypes [26]. The Catalonian NBS process is shown in Figure 1.

In addition, the method used for SCD screening detects the carrier status. Therefore, in 2021, the program took the initiative to inform families about children identified as carriers of the β^s^ and β^c^ alleles to offer genetic counseling and to incorporate this essential information into the child’s clinical record. This information was given by a pediatrician and a nurse specialist from the CRU via a telematic consultation in both prospective and retrospective cases. After that, a written report was issued and sent to the family and uploaded to the electronic health record. Presential consultation in the CRU is offered for families that express doubts or prefer face-to-face interaction due to language barriers or other reasons.

### 2.2. Impact of the NBS Program

To evaluate the impact of NBS on patients, we performed a retrospective questionnaire to collect demographic and clinical event information at clinical centers in Catalonia that follow SCD-affected children. Data were collected for patients in two cohorts, those diagnosed before (unscreened group, UG) and after (screened group, SG) NBS was introduced. Thirteen centers were included in the study, the CRU at Hospital Universitari Vall d’Hebron (Barcelona); 11 regional hospitals (Hospital Arnau de Vilanova de Lleida, Hospital de Figueres, Hospital Germans Trias i Pujol de Badalona, Hospital de Granollers, Hospital de Mataró, Hospital Mútua Terrassa, Hospital Santa Caterina de Salt, Hospital Sant Joan de Déu de Barcelona, Hospital de la Santa Creu i Sant Pau de Barcelona, Hospital de Reus, and Consorci Sanitari de Terrassa); and a primary care center in Vic. These centers collected data from all their SCD patients in both the UG and SG group.

We included all patients with a confirmed diagnosis of SCD. Data for the SG span from the initiation of the screening program from 2015 to 2021, both inclusive, and in the UG from 2001 to when screening began. The study incorporated 195 patients, 95 in the UG, 95 in the SG, and a further five patients identified through the NBS pilot program, which for calculation purposes were included in the SG.

We asked each center to retrospectively complete two forms for each patient, one relating to demographic data and the other to clinical events occurring within the first six years of life.

The demographic data form contained the following variables: diagnosis by NBS program (yes/no), date of birth, SCD genotype, date of diagnosis, start and end dates of follow-up, penicillin prophylaxis (yes/no), date of initiation of penicillin prophylaxis, treatment with hydroxyurea (yes/no), date of initiation of hydroxyurea treatment, neutropenia or other adverse effects to hydroxyurea (yes/no), bone marrow transplantation (yes/no), and date of bone marrow transplantation. We also included space for free-form comments.

The second form included the most common SCD-related clinical events in SCD pediatric patients in Spain [27], which were defined as VOCs, ACS, infections of probable bacterial origin (along with the identified microorganism) (BI), acute anemia requiring transfusion (TRF), acute splenic sequestration (ASSC), pathological transcranial Doppler echography (PTDE), and other non-SCD-related events. We also recorded whether the patient had to visit the emergency department (ER) and whether hospitalization was required for each event.

We calculated the time intervals from birth (in years) based on the information gathered, including the time until the first visit with a pediatric hematologist, the time until SCD diagnosis, the time until the initiation of penicillin prophylaxis, and the time until starting hydroxyurea treatment.

We compared the two cohorts using data from the first six years of patient follow-up. We looked at the number of major SCD-related events, ER visits, and hospitalizations per year of follow-up. Furthermore, we analyzed the impact of hydroxyurea treatment for each clinical event within both cohorts and assessed the age at which the first clinical event occurred.

### 2.3. Statistical Analysis of Questionnaire Data

We analyzed the categorical and continuous variables from the questionnaires in the second part of the study using SPSS v23 (IBM, Armonk, NY, USA) and GraphPad Prism v10.1 (GraphPad Software, La Jolla, CA, USA). We used non-parametric Kruskal–Wallis and Mann–Whitney U tests to compare continuous data across multiple groups and performed parametric analysis using Student’s *t*-test where applicable. We used Chi-squared tests to compare proportions.

We calculated Kaplan–Meier survival estimates (and 95% confidence intervals, CI) for remaining free from specific SCD-related complications in the first six years of life in the SG versus the UG. We compared the survival curves of the different events using the log-rank test. We considered a *p*-value less than 0.05 as statistically significant in all cases.

## 3. Results

### 3.1. Incidence and Epidemiology of SCD in Catalonia

As part of the NBS program of Catalonia, 506,996 newborns were screened for SCD from 2015 to 2022, both inclusive. The median age at sample collection was 48 h and 162 cases with the SCD phenotype detected by CE were referred to the CRU. All families were successfully contacted and attended the first appointment. Of those cases, 154 cases were confirmed by high-performance liquid chromatography (HPLC) and genetic analysis, four were confirmed by HPLC alone, and four cases resulted in false positive (FP) results (Table 1). Moreover, two false negative (FN) cases were diagnosed during this period. In total, 160 cases of SCD were diagnosed, corresponding to an incidence of 1 in 3169 newborns. Genetically, 64.4% (103 cases) presented the HbSS genotype, 25.6% HbSC (41 cases), and 7.5% HbSβ-thalassemia (3.8% β^s^/β^0^ and 3.8% β^s^/β^+^, six cases each). In 2.5% (four cases), the genotype was not available; in three of these cases the phenotype was suggestive of HbSS and in one, it was suggestive of HbSβ-thalassemia (β^s^/β^+^). Therefore, the sensitivity of the NBS program was 98.8%, the specificity was 99.9%, the positive predictive value was 97.5%, and the negative predictive value was 99.9%. The global accuracy was 99.9%.

Regarding the FP cases, one involved compound heterozygosity for HbS/Hb-Hope and another presented heterozygosity for a non-previously described alpha chain variant, which migrated to the S zone of the electropherogram. A third case exhibited a β^s^ allele in combination with a variant of unknown significance (VUS) initially described as a beta thalassemia mutation. Despite the initial classification, it clinically behaved as a carrier and was subsequently reclassified. The last FP case involved a sickle cell trait carrier and a gamma thalassemia. As for the FN cases, one of the two involved unreported multiple red blood cell transfusions, initially flagged as a sickle cell trait carrier, whereas the actual genotype was confirmed as HbSβ-thalassemia (β^s^/β^0^). This case was diagnosed through routine laboratory analysis. The second FN case was missed due to a human error during result validation, yet on inspection of the original data, the CE had indeed correctly identified it as HbSC. This case was diagnosed from the presentation of clinical symptoms (bone pain) at the age of two.

We examined the geographical origin of the mothers of newborns diagnosed with SCD. Of the total, 76.3% were from sub-Saharan Africa, 11.3% were from Central America, 8.1% were from North Africa, 3.8% were from South America, and 0.6% were from Spain. The distribution of paternal origin was similar. The predominant genotype among all origins was HbSS, except for the Spanish origin, where the sole patient presented a β^s^/β^0^ genotype, with the β^s^ allele inherited from the father, of Central American origin, and the β^0^ allele from the mother.

We also detected other hemoglobinopathies apart from SCD during the study period, which were 16 cases of hemoglobin C disease (1 in 31,687), six cases of HbD disease (1 in 84,499), six cases of beta thalassemia major (1 in 84,499), four cases of HbH disease (1 in 126,749), three cases of gamma thalassemia (1 in 168,999), one case of HbE disease (1 in 506,996), and one case of combined HbC and HbD disease (1 in 506,996).

Regarding carriers, we identified 3683 β^s^ allele carriers (1 in 138), 927 β^c^ allele carriers (1 in 546), 179 β^D^ allele carriers (1 in 2832), and 195 β^E^ allele carriers (1 in 2600). In total, there were 4984 carriers, which corresponds to an incidence of 1 in 102 newborns in Catalonia across the study period. This incidence has progressively increased annually from 0.84% in 2015 to 1.08% in 2022, reaching its peak in 2021 with a 1.12% incidence.

### 3.2. Clinical Impact of the NBS Program

#### 3.2.1. Demographic Data of the Study Cohort

The median follow-up time was 3.58 y (years) in the SG and 8.67 y in the UG. Considering that the maximum follow-up for the SG is limited to six years since the start of the NBS program, we only considered events in the UG up to six years old. As a result, the adjusted median follow-up times for up to six years old for the SG and UG were 3.58 y and 4.46 y, respectively (*p* = 0.02). The total years of follow-up during the first six years of life were 339.76 for the SG and 399.35 for the UG. The median age at diagnosis was significantly younger in the SG compared to the UG (0.1 y vs. 1.68 y, *p* < 0.0001), as detailed in Table 2. During the study period, three patients from the SG and two patients in the UG underwent hematopoietic stem cell transplantation (HSCT). These patients presented the HbSS genotype with severe or recurrent acute complications and a match sibling donor available. The genotype information was available for all patients, except for one individual in the SG. There were statistically significant differences (*p* = 0.025) in the proportions of these genotypes between the two cohorts. The proportions of SCD genotypes in the SG population were 61% HbSS, 26% HbSC, 5% HbSβ^0^, 7% HbSβ^+^, and in 1%, the genotype was not available. In the UG, 77.9% were HbSS, 13.7% HbSC, 7.4% HbSβ^0^, and 1.1% HbSO-Arab.

Penicillin prophylaxis was initiated in nearly all cases in both the SG and UG (100% vs. 94.7%, *p* = 0.03), however, it was introduced at a significantly younger median age in the SG (0.12 y vs. 1.86 y, *p* < 0.0001). We selected only the HbSS and HbSβ^0^ genotypes for inclusion in hydroxyurea treatment calculations since this treatment is only indicated for these genotypes. Fewer patients in the SG were reported to have received hydroxyurea (80.3% vs. 93.8%, *p* = 0.013). In the SG, hydroxyurea treatment was not initiated in 13 cases, one case where the family refused treatment; two cases where follow-up was lost after diagnosis; six cases where the patients were less than one year old, so they had not reached the age required to begin treatment; two patients who started hydroxyurea treatment after the study data collection finished; and in the other two cases, the pediatric hematologists decide not to initiate hydroxyurea due to good general clinical condition in the patients. The SG members were also significantly younger when starting hydroxyurea treatment (1.42 y vs. 4.5 y, *p* < 0.0001). We observed no significant differences in hydroxyurea-related neutropenia or related adverse effects between the cohorts (Table 2).

#### 3.2.2. Clinical Events during the First Six Years of Life

The patients in the SG experienced their first clinical event earlier (1.04 y vs. 1.64 y, *p* = 0.035), as well as their first SCD-related event (1.14 y vs. 1.91 y, *p* = 0.046). This was observed across all subtypes of SCD clinical events, but only remained statistically significant for VOCs and TRFs. (Table 2).

**Figure 2:**
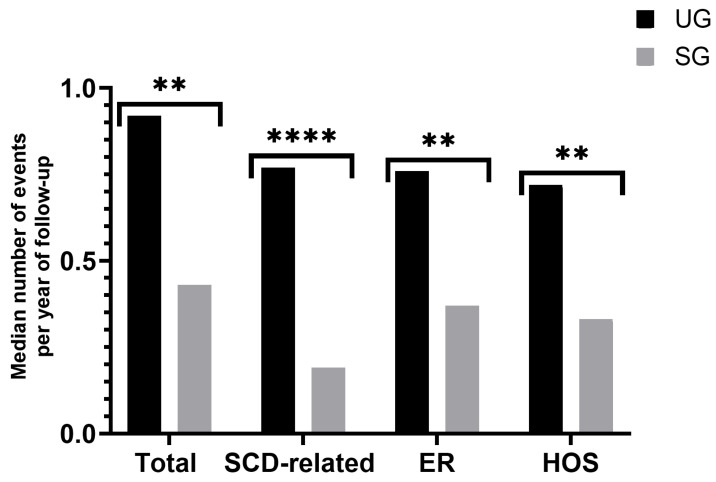
Clinical events by year of follow-up. SG: screened group; UG: unscreened group; Total: total clinical events; SCD-related: sickle cell disease-related clinical events; ER: visits to the emergency department; HOS: hospitalizations. The level of statistical significance is indicated by asterisks: ** *p* < 0.001; **** *p* < 0.00001.

**Figure 3:**
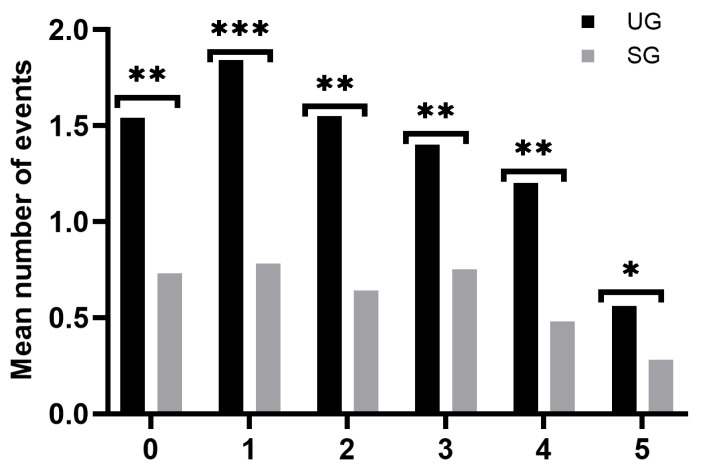
Mean number of SCD-related clinical events by year of age in the study cohorts. SG: screened group; UG: unscreened group. The level of statistical significance is indicated by asterisks: * for *p* < 0.05, ** for *p* < 0.01, and *** for *p* < 0.001.

There was one reported death in the study, corresponding to a patient in the SG, due to a Salmonella enterica infection at the age of two. We observed at least one major documented SCD-related complication in 67.7% of all patients, while it was less frequent in the SG than in the UG (55.0% vs. 81.1%, *p* < 0.001). We also saw that patients in the SG experienced on average (median) fewer clinical events per year of follow-up (0.43 vs. 0.92, *p* < 0.001), which remained true when we excluded non-SCD-related events (0.19 vs.0.77, *p* < 0.00001). The mean number of VOCs per year of follow-up was half as frequent in the SG (0.2 vs. 0.41, *p* = 0.008), and ACSs happened just a quarter of the time (0.04 vs. 0.18, *p* < 0.001). The other SCD-related clinical events also showed slightly reduced occurrence frequencies but lacked statistical significance as follows: TRFs (0.14 vs.0.28, *p* = 0.05), BIs (0.12 vs. 0.15, *p* = 0.4), and ASSC (0.01 vs. 0.05, *p* = 0.02). The number of PTDE events recorded was not enough to perform calculations. Patients in the SG recorded fewer visits to the ER (0.37 vs. 0.76, *p* < 0.0001) and fewer hospitalizations (0.33 vs. 0.72, *p* < 0.0001) (Figure 2). The mean number of clinical events per year of life was significantly lower in the SG for all ages (Figure 3).

**Figure 4:**
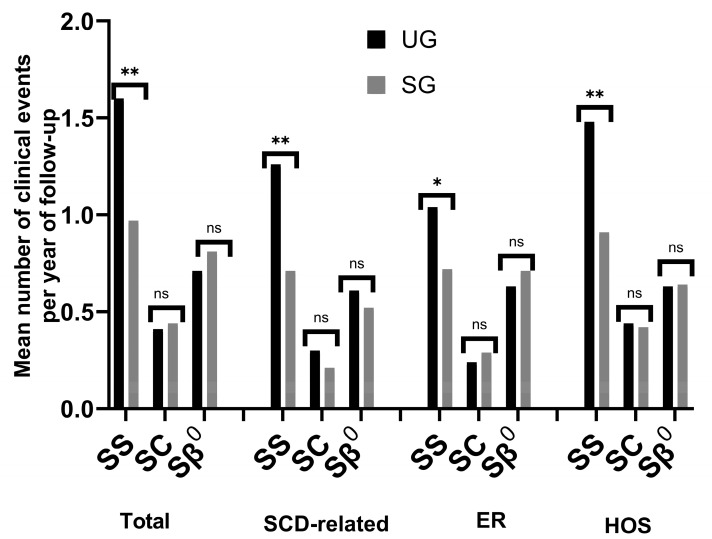
Clinical events by genotype in the study cohorts. SG: screening group; UG: unscreened group; Total: total clinical events; SCD-related: sickle cell disease-related clinical events; ER: visits to the emergency department; HOS: hospitalizations. The level of statistical significance is indicated by asterisks: * *p* < 0.05; ** *p* < 0.01, ns (not significant). Genotypes: SS (HbSS); SC(HbSC); Sβ^0^(HBSβ^0^).

The SCD genotype influenced the frequency of clinical events, and individuals with HbSS experienced more clinical events, ER visits, and hospitalizations than the other genotypes. This was particularly marked in the UG. There was a statistically significant difference in the mean number of clinical events, ER visits, and hospitalizations between the SG and UG. After HbSS, the HbSβ^0^ group showed the second-highest frequency of events across all categories, followed by HbSC. Intriguingly, the HbSβ^0^ and HbSC genotype subgroups displayed similar average numbers of clinical events, ER visits, and hospitalizations, irrespective of whether the patients underwent screening (Figure 4).

**Figure 5:**
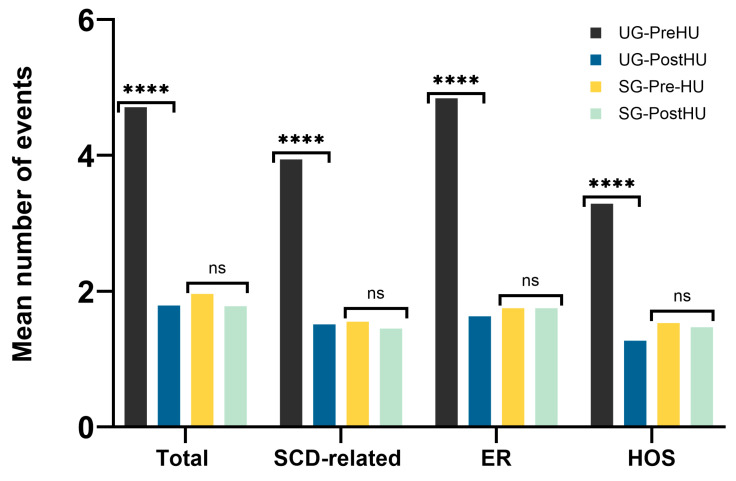
Impact of hydroxyurea treatment on the events in both the UG and SG. UG-PreHU: unscreened group pre-hydroxyurea; UG-PostHU: unscreened group post-hydroxyurea; SG-PreHU: screened group pre-hydroxyurea; SG-PostHU: screened group post-hydorxyurea; Total: total clinical events; SCD-related: sickle cell disease-related clinical events; ER: visits to the emergency department; HOS: hospitalizations. The level of statistical significance is indicated by asterisks: **** *p* < 0.0001, ns (not significant).

Receiving treatment with hydroxyurea in the UG displayed a drastic reduction in all clinical events, including visits to the ER and hospitalizations. However, in the SG, we did not observe any significant difference in clinical events if hydroxyurea was used (Figure 5).

**Figure 6:**
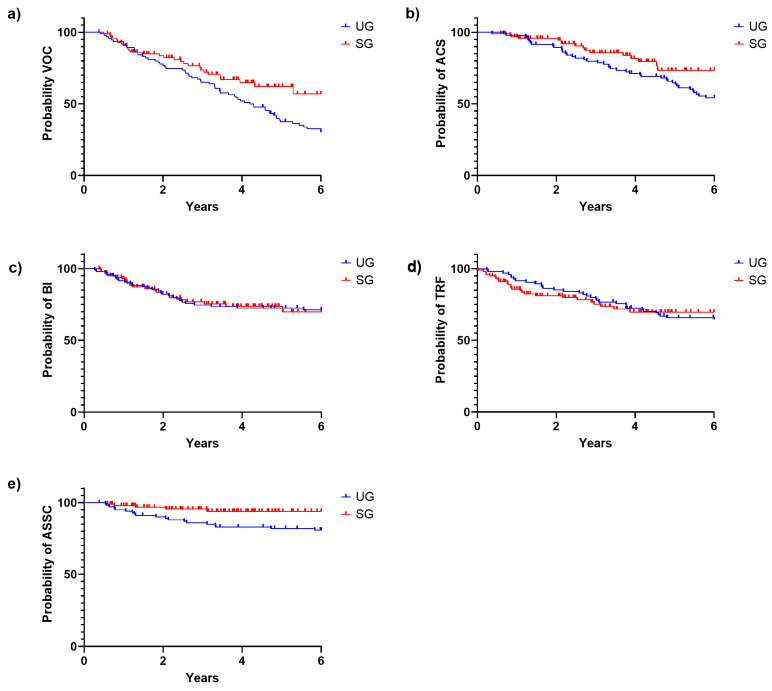
Kaplan–Meier curves for event-free survival by specific events in the SG and UG cohorts. Each graph represents the six-year survival estimate since birth without the corresponding SCD-related event. SG: screened group; UG: unscreened group. (**a**) Six-year Kaplan–Meier estimate without vaso-occlusive crisis (VOC) was different in both groups (57.0% vs. 30.3%, *p* = 0.03). (**b**) Six-year Kaplan–Meier estimate without acute chest syndrome (ACS) was not different in both groups (73.5% vs. 54.3%, *p* = 0.06). (**c**) Six-year Kaplan–Meier estimate without infections of probable bacterial origin (BI) was not different in both groups (71.3% vs. 69.8% *p* = 1.0). (**d**) Six-year Kaplan–Meier estimate without infections of probable acute anemia requiring transfusion (TRF) was not different in both groups (64.5% vs. 69.7%, *p* = 0.9). (**e**) Six-year Kaplan–Meier estimate without acute splenic sequestration (ASSC) was not different in both groups (93.8% vs. 85.0%, *p* = 0.12).

Following the Kaplan–Meier analysis, we saw that the six-year survival estimate without VOCs was higher in the SG than in the UG (57.0% vs. 30.3%, *p* = 0.03). No significant differences were found for ACS (73.5% vs. 54.3%, *p* = 0.06), BIs (71.3% vs. 69.8% *p* = 1.0), TRFs (64.5% vs. 69.7%, *p* = 0.9), and ASSC (93.8% vs. 85.0%, *p* = 0.12). There were not enough data to calculate the differences in the case of PTDE (Figure 6).

## 4. Discussion

This study demonstrates the impact of including SCD in the Catalonian NBS program during the first eight years. SCD has emerged as the second most prevalent disease detected through the NBS program with an incidence of 1 in 3169 (and increasing), second only to congenital hypothyroidism (1 in 2241) [18]. Between 2015 and 2022, 160 newborns were diagnosed with SCD, predominantly with the HbSS genotype and from parents of sub-Saharan African origin. Genetic diagnosis information was available in nearly all cases (97.5%), which differs from other NBS programs, which only perform genetic testing in case of doubt or to aid genetic counseling [10].

Catalonia is the Spanish region with the highest number of foreign inhabitants, and in 2023 had over 300,000 individuals of African descent and nearly 100,000 people from South and Central America, according to the Spanish National Statistics Institute (INE) [28]. While Catalonia has experienced a 21% decrease in the total birth rate over the last decade, there has been a 6% increase in mothers of foreign origin [29]. This migration has been a strong driver for dispersing β^s^ allele carriers globally, particularly to North America and Western Europe [30].

The incidence of SCD in Catalonia surpasses regions with similar demographic characteristics in Spain, such as Madrid (1 in 5552) [12] and Murcia (1 in 10,000) [31]. However, it is lower than in some regions of Belgium where NBS for SCD is available (Brussels and Liege) (1 in 1427) [11], in England (1 in 2564) [32], in a targeted population in France (1 in 1303) [14], or the USA (1 in 1941) [33]. Data reported from some pilot studies in Germany (1 in 3688) [10] show a similar incidence to that which we see in Catalonia. The incidence rate obtained in the pilot study before starting the NBS program in Catalonia was higher, at 1 in 2697 (data not previously published). This difference might be attributed to potential bias in the selected population in the pilot study, since only approximately one-third of Catalan newborns were included.

The Catalonian NBS extended its scope to include additional hemoglobinopathies and to detect β^S^ and β^c^ allele carriers. This follows the European Consensus for NBS from 2017, which advocates for universal screening, carrier detection, and reporting of beta thalassemia major [34]. This expansion has allowed for the early intervention and monitoring of children affected by severe conditions, especially beta and alpha thalassemia major. Since the NBS program’s inception in 2015, the detection of newborn carriers of the sickle cell trait has increased, with an incidence of 1 in 138 for Hb S carriers and 1 in 547 for Hb C carriers. This surpasses that of Madrid but falls considerably short of rates in other European countries [12].

As for the technical methodology use, the Catalonian NBS program uses CE, a recommended technique by the European Consensus for NBS [34]. We confirm its relevance by the high diagnostic accuracy achieved in this study. The sensitivity, specificity, and positive and negative predictive value metrics show slightly superior performance compared with other NBS programs, including Madrid, which uses HPLC [12], and England, which uses CE, HPLC, tandem mass spectrometry, and isoelectric focusing [32].

Of the two FN cases detected here, the first emphasizes the need to take a second sample months after finishing the last blood transfusion (as per international guidelines) [35]. The second FN was caused by human error, and a reprogramming of the laboratory software to highlight positive results was developed, aiming to reduce this possibility. There was a low but non-zero frequency (2.5%) of FP cases, as previously observed for CE and HPLC [36]. This also highlights the need for confirmatory testing of all cases, such as by the near universal genetic analysis performed here in the Catalonian NBS.

The study’s second objective was to assess how NBS has impacted the clinical events of SCD patients. The median age at diagnosis significantly differed between the SG (0.1 y) and the UG (1.7 y), comparable to what was observed in the Belgium and Quebec NBS programs, with 0 y for the SG and 1 y in the UG in both studies [19,37]. In Catalonia, the NBS program collects samples within 48–72 h of birth, with most positive cases referred to the CRU within a week. The initial visit to the CRU occurs at two months, aligning with SEHOP and international guidelines [25]. In contrast, the UG’s diagnosis typically occurs at around two years, correlating with their first reported clinical event. The median age at the first event was consistently lower in the SG for all assessed clinical events. This trend, akin to the Belgian experience [19], likely stems from closer monitoring of the SG and potential underreporting of clinical events in the UG before diagnosis.

The European Consensus for NBS states that any SCD NBS implementation should incorporate measures to mitigate morbidity and mortality, such as anti-pneumococcal penicillin prophylaxis, vaccination, comprehensive follow-up, and parental education [34]. The impact of SCD NBS on mortality has been widely studied. In Jamaica, they witnessed a decrease in mortality from 14% to less than 1% when preventive strategies were introduced at an early age [38]. In Dallas, they also reported a decline in mortality among screened individuals [39], and several other NBS programs have demonstrated the advantages of implementing national or regional programs for SCD [40,41,42].

In our study, we observed just one death in the SG and no deaths in the UG. This low mortality is likely related to the high standards of follow-up and treatment for these patients in Spain, a high-income country. The cause of death in our study was attributed to *Salmonella enterica*, which is not currently covered by routine vaccinations for SCD patients. This sparks a discussion as to its potential inclusion in vaccination programs. However, there is a lack of randomized controlled trials evaluating the efficacy and safety of salmonella vaccines in this population and further research is needed [43].

The impact of NBS on patient morbidity has previously been far less explored. Our study is the first to compare two large cohorts of patients across multiple clinical events, before and after the implementation of NBS. Our findings indicate that NBS reduced the median number of clinical events per year of follow-up by 50%, decreased SCD-related events by 75%, and halved the frequency of visits to the ER and hospitalizations. These results are in concordance with the recently published experience in Quebec [37]. There is also a clear difference in the pattern of clinical events. In the SG, patients are diagnosed in the first two months of life and experience a lower clinical event frequency that remains stable, in contrast to the UG with a much higher overall clinical event occurrence frequency that peaks in the second year of life, when most individuals in this group are diagnosed, before slowly declining.

Our study concurs with existing studies showing that VOC and ACS are the most prevalent clinical events. However, ASSC occurs at a comparatively lower frequency in our case [20,44]. The six-year Kaplan–Meier survival estimates reveal distinctions between the cohorts in the likelihood of survival without VOCs but not for ACS, BIs, TRFs, or ASSC. These outcomes contrast with those from the Belgium cohort, where they only observed that the 15-year Kaplan–Meier estimate without bacteremia was significantly higher in the screened group than the unscreened group [19].

Prior studies have suggested clinical indistinguishability between HbSS and HbSβ^0^ phenotypes [45], whereas we observed more clinical events in HbSS-affected individuals. This discrepancy could be attributed to the smaller sample size of HbSβ^0^ patients. As is typical, the HbSC phenotype demonstrates a lower frequency of clinical events compared to HbSS and HbSβ^0^ cases [46]. The HbSC phenotype often manifests as different conditions, such as retinopathy and osteonecrosis, with delayed symptom onset. The reduced frequency of clinical events in the HbSC genotype may explain the differences in the number of diagnosed patients with this genotype between the two cohorts. Notably, NBS enables earlier diagnosis, before symptom onset, and thus holds the potential to identify more HbSC patients and alleviate complications that could result from a delayed diagnosis.

In 1986, a randomized trial of penicillin administration in children with SCD under three years old demonstrated a reduction in the morbidity and mortality associated with pneumococcal septicemia, providing a clear justification for NBS for SCD [47]. This was a proof of concept as to how early detection can modify disease evolution. The use of hydroxyurea treatment has sparked debate due to concerns about its toxicity. However, the BABY-HUG clinical trial demonstrated significant clinical benefits, reducing pain and dactylitis, and showed promise in lowering ACS, hospitalizations, and transfusions. Notably, there were no effect on ASSC or impact on growth or neurodevelopment, with mild-to-moderate neutropenia being the only reported side effect [48].

In Catalonia, hydroxyurea treatment is offered to both symptomatic and asymptomatic patients starting from nine months old. In our study, there was a significantly lower proportion of patients receiving hydroxyurea in the SG compared to the UG. NBS leads to a much earlier diagnosis of patients in the first nine months of life, before hydroxyurea treatment is recommended. Most cases where hydroxyurea was not administered in this study were due to the young age of the participants or the attending physicians still deciding whether to start it. The results were further skewed by some patients lost to follow-up after diagnosis in the SG group. This explains the seemingly paradoxical situation where early diagnosis led to less treatment.

A potential further bias could stem from changes in the clinical guidelines during the early phase of the study period. In 2014, before NBS started, following the BABY-HUG [26] study and JAMA recommendations [49], we introduced the policy of starting hydroxyurea treatment between 9 and 12 months old. Before 2014, the guidelines did not recommend starting hydroxyurea before the age of two. This could theoretically reduce the number of treated patients in the UG. However, the potential bias is minimized, since the median diagnosis age of patients in the UG was 1.68 years.

The incidence of hydroxyurea-related neutropenia in our study aligns with findings from other studies, emphasizing the drug‘s favorable safety profile [50,51]. We found that hydroxyurea demonstrated a distinct impact on the UG, with a notable decrease in the average number of clinical events, ER visits, and hospitalizations. However, in the SG, the observable effect of hydroxyurea is less pronounced. This may be due to the effectiveness of the early introduction of hydroxyurea, at nine months old, which results in SCD-related events as infrequent as the low number of events typically experienced by patients before turning one year old. It is noteworthy that in our study, hydroxyurea did not significantly impact the incidence of ASSC, an area of concern for hydroxyurea treatment [52]; in fact, in both cohorts, it reduced the incidence of ASSC. Recently, Allali et al. indicated that early intervention with hydroxyurea may contribute to the prolonged preservation of spleen perfusion and function. This could potentially delay the onset of ASSC, which occurs at an unusually advanced age in patients undergoing hydroxyurea treatment [53]. Our findings support hydroxyurea as a safe intervention to reduce the onset of SCD-related clinical events with few side effects.

The primary limitation of our study revolves around potentially incomplete data in the UG. This may arise from clinical events predating the SCD diagnosis, possibly leading to misdiagnoses or omissions in the clinical records. Additionally, language and socioeconomic barriers might have led to underreporting of clinical events. Future studies should seek to minimize these issues and understand their impact on the reported clinical events. We acknowledge that a prospective study could provide clarity to some of the potential biases mentioned above with our retrospective study. However, we consider that a prospective study with a cohort having to forego NBS is not ethical for patients when this retrospective data are available. The retrospective approach maintains ethical integrity and generates equity. Extending this retrospective approach to other regions, however, must be performed with caution and with consideration of the different clinical practices used in those regions before and after screening was introduced, as well as other potential biases.

## 5. Conclusions

Implementing NBS for SCD in Catalonia has allowed us to calculate the incidence of SCD and other hemoglobinopathies in Catalonia, including carrier status. The introduction of SCD NBS has empowered us to take preventive measures to reduce the occurrence of clinical events and provide genetic counseling education to improve patient outcomes. This comprehensive program, which includes laboratory-based detection and genetic confirmation, antibiotic prophylaxis, vaccination, early introduction of hydroxyurea treatment for the most severe genotypes, family education on SCD management, and a well-established network with regional hospitals for ongoing care, has demonstrated its efficacy by substantially reducing the most frequent events related to SCD during childhood.

## Figures and Tables

**Table 1:** Screening results for SCD and confirmed diagnosis groupings.

	Confirmatory Diagnosis by Genetic Analysis (Sanger and MLPA if Necessary)							
Phenotype by CE	HbSS	HbSβ^0^	HbSβ^+^	HbSC	HbSA ^Ϯ^	HbSX ^Ϯ^	NA	*n*
FS	103 (89.6%)	2 (1.7%)	5 (4.3%)		1 (0.9%)	1 (0.9%) *	3 (2.6%)	115
FSa		3 (42.9%)	1 (14.3%)		1 (14.3%)	1 (14.3%) ^#^	1 (14.3%)	7
FSC				40 (100%)				40
FAS ^Ω^		1 (100%)						1
FSC ^Ω^				1 (100%)				1
							Total	164

Results are expressed as total number of cases diagnosed and percentage (%). ^Ϯ^ False positive cases. ^Ω^ False negative cases. CE: capillary electrophoresis; NA: not available. MLPA: multiplex ligation-dependent probe amplification. HbSX: * HbS/Hb-Hope; ^#^ Hb + α-chain variant. HbSA: HbS/HbA. Phenotype by CE (fractions are ordered based on their proportion on the electropherogram). FS: fetal hemoglobin + hemoglobin S; FSa: fetal hemoglobin + hemoglobin S + hemoglobin A; FSC: fetal hemoglobin + hemoglobin S + hemoglobin A; FAS: fetal hemoglobin + hemoglobin A + hemoglobin S.

**Table 2:** Demographic characteristic and clinical characteristic comparison between the unscreened group (UG) and NBS group (SG).

	UG	SG	*p*
N	95	100	
Median time of follow-up (y)	8.67 (0.66–18.2)	3.58 (0.25–8.1)	*p* < 0.0001 *
Median time of follow-up, up to six years of life (y)	4.46 (0.66–6)	3.58 (0.25–6)	*p* = 0.02 *
Total years of follow-up (up to six years of life)	399.35	340.76	
Deaths	0	1	
HSC transplantation	2	3	
Median age at diagnostics (y) (range)	1.68 (0.1–5.69)	0.1 (0–1.99)	*p* < 0.0001 *
Median age at last follow-up (y) (range)	10.43 (1.48–20.97)	3.79 (0.38–8.2)	*p* < 0.0001 *
ATB prophylaxis (%)	94.7	100	*p* = 0.03 *
Median age at ATB onset, (y) (range)	1.86 (0–11.42)	0.12(0.01–4.44)	*p* < 0.0001 *
HU treatment (%)	93.8	80.3	*p* = 0.013 *
Median age at HU onset, (y) (range)	4.5 (1.23–11.91)	1.42 (0.76–3.96)	*p* < 0.0001 *
HU-related neutropenia (%)	6.6	9.4	*p* = 0.21
HU-related adverse effects (%)	5.3	5.7	*p* = 0.1
Age at first VOC (y)	2.96 (0.4–5.7)	1.91 (0.5–5.3)	0.007 *
Age at first ACS (y)	3.33 (0.7–5.8)	2.58 (0.4–4.6)	0.074
Age at first BI (y)	1.80 (0.3–5.6)	1.22 (0.4–5.0)	0.52
Age at first TRF (y)	2.72 (0.2–6)	0.85 (0.1–3.4)	0.001 *
Age at first ASSC (y)	1.98 (0.6–5.8)	1.30 (0.5–3.1)	0.355

N: sample size; y: year; HSC: hematopoietic stem cell; ATB: antibiotic (penicillin); HU: hydroxyurea; VOC: vaso-oclusive crisis: ACS: acute chest syndrome; BI: bacterial infection; TRF: acute anemia requiring transfusion; ASSC: acute splenic sequestration. * *p*-values below 0.05 are considered statistically significant. Data are shown as median, range, and *p*-values.

## Data Availability

The data that support the findings of this study are available from the corresponding author upon reasonable request.
